# Genomic analyses detect Eurasian-lineage H10 and additional H14 influenza A viruses recovered from waterfowl in the Central United States

**DOI:** 10.1111/irv.12250

**Published:** 2014-04-02

**Authors:** Anthony C Fries, Jacqueline M Nolting, Andrew S Bowman, Mary L Killian, David E Wentworth, Richard D Slemons

**Affiliations:** a Department of Veterinary Preventive Medicine, The Ohio State UniversityColumbus, OH, USA; b Department of Evolution, Ecology and Organismal Biology, The Ohio State UniversityColumbus, OH, USA; c National Veterinary Services Laboratories, USDA, APHISAmes, IA, USA; d Virology, J. Craig Venter InstituteRockville, MD, USA

**Keywords:** H10 subtype, H14 subtype, influenza A virus, migratory birds, surveillance

## Abstract

The accurate and timely characterization of influenza A viruses (IAV) from natural reservoirs is essential for responses to animal and public health threats. Differences between antigenic and genetic subtyping results for 161 IAV isolates recovered from migratory birds in the central United States during 2010–2011 delayed the recognition of four isolates of interest. Genomic sequencing identified the first reported Eurasian-origin H10 subtype in North America and three additional H14 isolates showing divergence from previously reported H14 isolates. Genomic analyses revealed additional diversity among IAV isolates not detected by antigenic subtyping and provided further insight into interhemispheric spread of avian-origin IAVs.

## Introduction

The timely detection and characterization of novel or emerging avian-origin influenza A viruses (IAV) is crucial to prepare for health threats these viruses pose to humans and animals. All modern IAV pandemics have contained genetic segments derived from avian-origin IAVs.^1^ Avian-origin IAVs are prone to nucleotide mutations and undergo frequent genomic reassortment of their single-stranded, segmented RNA genome resulting in genetically and antigenically diverse assemblages.^2^ Distinguishable eastern and western hemispheric lineages exist in avian-origin IAV gene segments due to geographic separation of natural hosts.^3^ Monitoring for emergent IAV strains is based on the characterization of the antigenic properties of surface proteins and by genomic sequencing.

Ongoing avian-origin IAV surveillance in the Mississippi Migratory Bird Flyway from July 2010 to January 2011 resulted in 161 IAV isolates recovered from 2879 cloacal swabs collected from migratory waterfowl as previously described.^4^ We identified three H14 isolates by antigenic subtyping. Due to the rarity of the H14 subtype and recovery in the western hemisphere, those three isolates were immediately sequenced and characterized.^5^,^6^ As described below, the genomes of the additional 158 IAV isolates were subsequently sequenced, and analyses identified additional H14 and Eurasian-origin H10, N6, and NS genomic segments.

## Methods

Viral isolations were performed as previously described, and all IAV isolates were submitted to the USDA APHIS National Veterinary Services Laboratories (NVSL) for antigenic subtyping with HI/NI tests.^4^,^7^ After antigenic subtyping, isolates were sequenced using a next-generation sequencing pipeline at the J. Craig Venter Institute, which includes a Roche GS-FLX and an Illumina HiSeq 2000. Full nucleotide sequences for each isolate are available on GenBank: CY131984-CY132000, CY132085-CY132148, CY132638-CY133876, KC110594-KC110614, JN696314-JN696316. The genetic subtype of each isolate was determined based on amino acid (AA) similarity to published subtypes. PCR subtyping was not performed on the original samples prior to virus isolation or on first passage isolates. Open reading frames were used in phylogenetic analyses. The NCBI BLAST tool was used to obtain high percent identity sequences to each segment from public databases. RAxML 7.2.8^8^ was used for maximum-likelihood phylogeny construction using 1000 bootstrap replicates, and the best supported nucleotide model identified in ModelTest.^9^ Tree branch lengths are representative of the number of substitutions/site.

## Results and Discussion

After genomic sequencing of the 158 IAV isolates, we identified four isolates of interest including a Eurasian-origin H10 IAV and three additional H14 IAV isolates. Based on genomic sequencing, these isolates were named as follows: A/long-tailed duck/Wisconsin/10OS3919/2010(H10N6), A/long-tailed duck/Wisconsin/10OS3918/2010(H14N8), A/mallard/Wisconsin/10OS3941/2010(H14N6), and A/northern shoveler/Missouri/10OS4673/2010(H14N6).

### H10 virus

**Figure 1:**
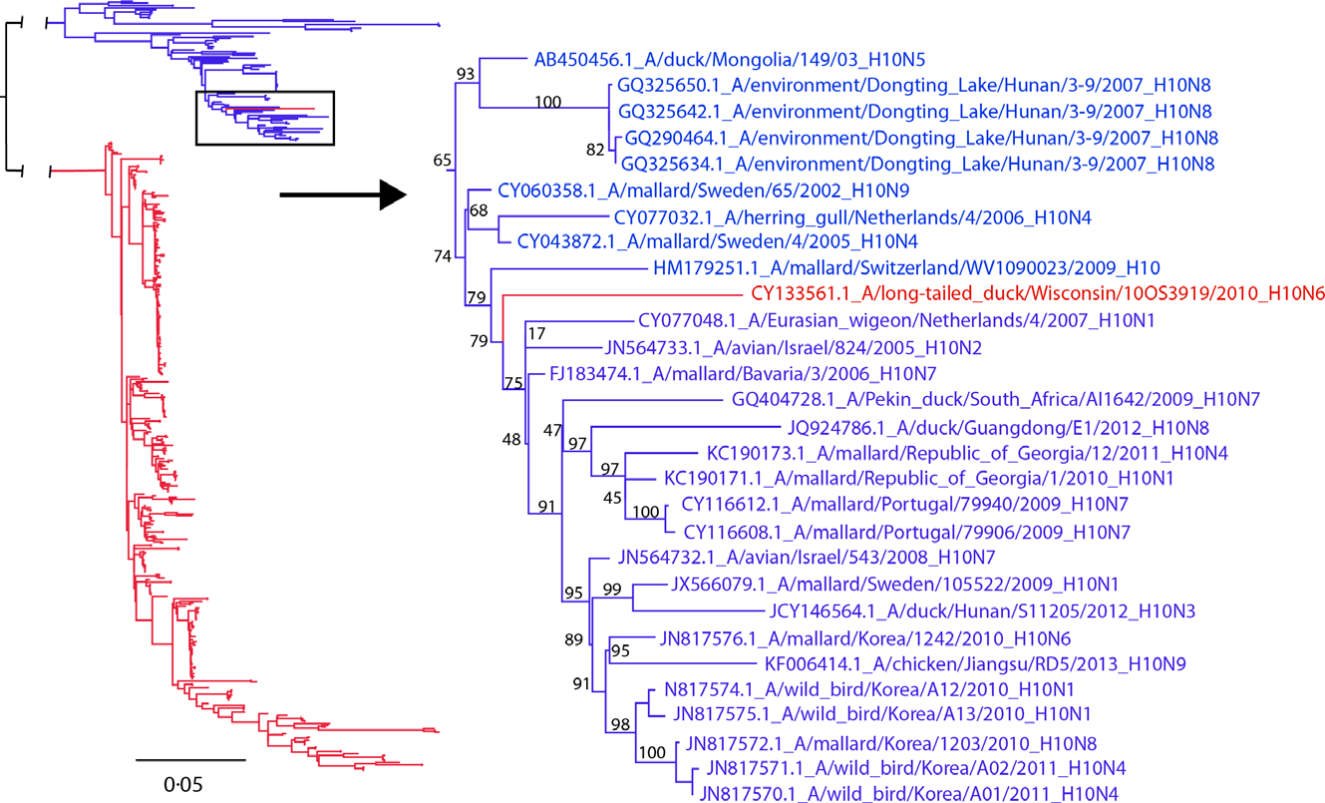
Maximum-likelihood tree with GTR+*Γ* nucleotide model for H10 hemagglutinin gene segments emphasizing placement of A/long-tailed duck/Wisconsin/10OS3919/2010(H10N6). Included are all North American (red) and Eurasian (blue) H10 segments publically available (*n* = 460).

Avian-origin IAVs are separated into Eurasian and American lineages.^3^ Genetic segments of either lineage are occasionally detected between geographic regions and when identified are infrequently detected in the interior of continents.^10^ Antigenic subtyping of A/long-tailed duck/Wisconsin/10OS3919/2010(H10N6) was inconclusive whereas genetically, the nucleotide sequence of the HA gene was consistent with interhemispheric gene flow of the Eurasian H10 IAV lineage into North America (Figure1). This is the first documentation of a Eurasian-lineage H10 IAV genomic segment in North America and is currently relevant given the H10 infections and deaths in humans from avian-origin H10 IAVs in China.^11^ Additionally, the relatively long branch length shown in Figure1 (0·041 substitutions/site, μ = 0·0025, σ = 0·0064 for terminal branches of all publically available H10, *n* = 460) indicates that the Eurasian-lineage H10 may have been circulating in North America for many years prior to this detection. Interestingly, a recent study found that the North American lineage of H10 has been circulating in Australia for several years.^12^ Further surveillance is needed in less monitored species such as sea ducks, from which this isolate was recovered, to determine whether Eurasian-linage H10 is maintained in niche reservoirs within North America.

**Figure 2:**
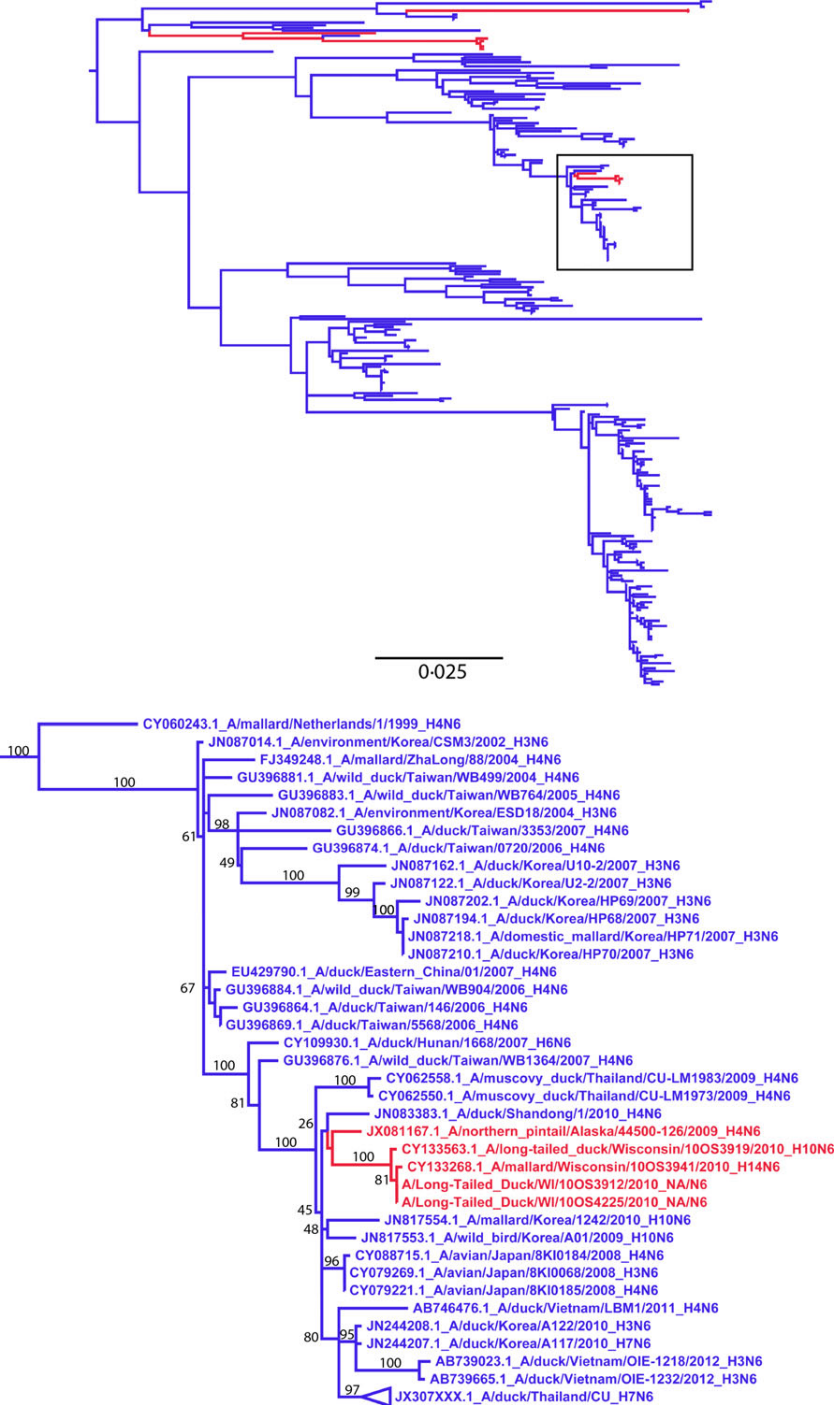
Maximum-likelihood tree with GTR+*Γ* nucleotide model for the N6 neuraminidase subtype segments. North American (red) and Eurasian (blue) segments were selected based on percent identity of the N6 segment of A/long-tailed duck/Wisconsin/10OS3919/2010(H10N6), and the 500 most similar sequences that were publically available.

The NA segment paired with this Eurasian-origin H10 segment showed 99·8% AA identity (469/470) to the previously reported Eurasian-origin N6 of the A/long-tailed duck/Wisconsin/10OS4225/2010(H14N6) and A/long-tailed duck/Wisconsin/10OS3912/2010(H14N6) [5] and to A/mallard/Wisconsin/10OS3941/2010(H14N6) whose sequence is detailed in this report (Figure2).

**Figure 3:**
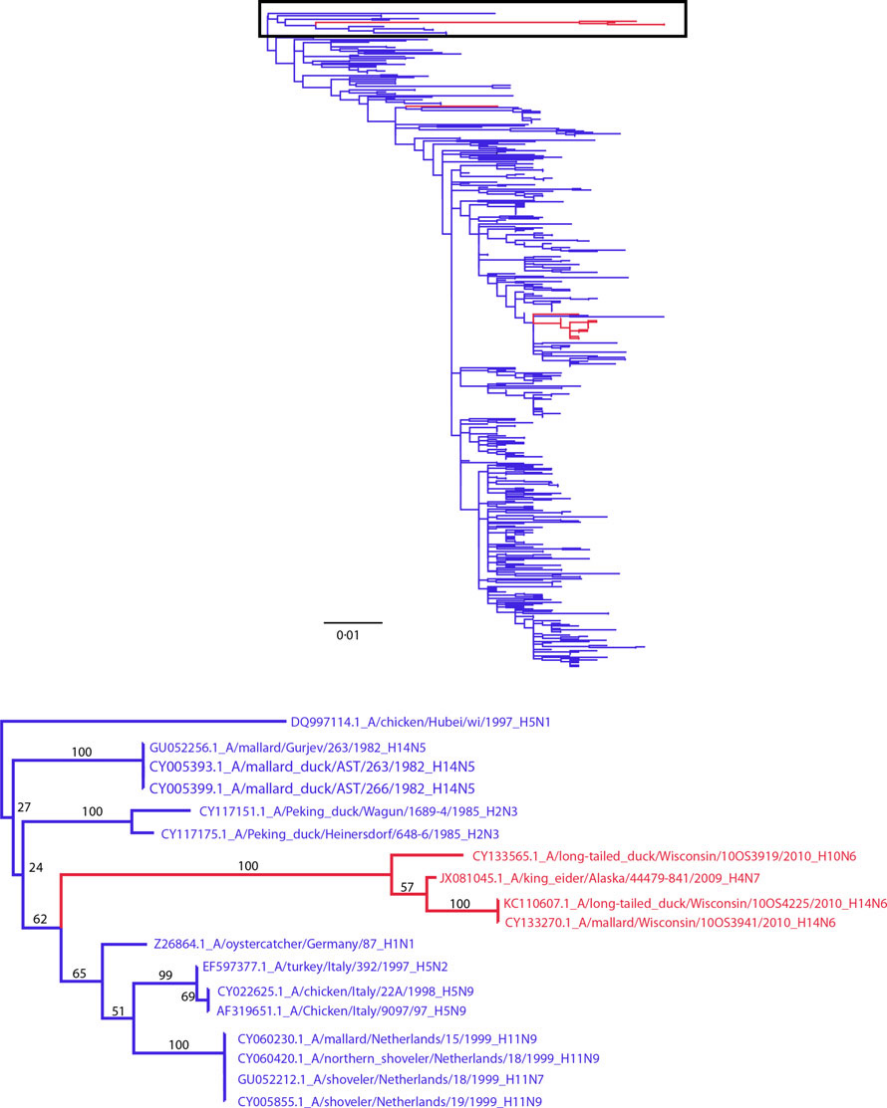
Maximum-likelihood tree with GTR+*Γ* model for the non-structural (NS) gene emphasizing the placement of the Eurasian-origin H10 and H14 isolates. North American (red) and Eurasian (blue) segments were selected based on the percent identity to the NS segment of A/long-tailed duck/Wisconsin/10OS3919/2010(H10N6), and the 500 most similar nucleotide sequences that were publically available.

In addition, the non-structural (NS) gene segment of A/long-tailed duck/Wisconsin/10OS3919/2010(H10N6) shared a 98·9% AA identity (275/278) with Eurasian-origin NS gene segments of the A/mallard/Wisconsin/10OS3941/2010(H14N6) described below and previously reported the A/long-tailed duck/Wisconsin/10OS4225/2010(H14N6).^13^ These NS segments shown in Figure3 represent a phylogenetically unique subclade that includes A/mallard/Gurjev/263/1982(H14N5) from 28 years prior.^13^ The branch to this clade was the longest branch on the NS tree at 0·045 substitutions/site and indicates that mutational intermediaries of this NS clade are rarely detected. The only other segment within this NS clade was recovered from another sea duck, A/King Eider/Alaska/44479-841/2009(H4N7), GenBank Accession No. JX081045. Previous studies have shown sea duck-origin IAVs can be divergent from both North American and Eurasian wild-bird IAVs^14^ indicating additional IAV genetic diversity that may be circulating in this under-sampled ecological niche.

### H14 viruses

Genomic sequencing revealed three additional H14 isolates that were not identified using the antigenic subtyping protocols that detected the three H14 IAV isolates detailed in a previous report.^5^ The antigenic subtyping results for two of the three isolates (A/long-tailed duck/Wisconsin/10OS3918/2010(H14N8)) and (A/mallard/Wisconsin/10OS3941/2010(H14N6)) were inconclusive. These inconclusive results are surprising because the HA segments of these two isolates had AA sequences identical to the previously reported H14 isolates from Wisconsin which were detected antigenically.^5^

The third H14 isolate, A/northern sвhoveler/Missouri/10OS4673/2010(H14N6), was antigenically characterized as an H4 subtype. Phylogenetically, H4 is most closely related to H14^6^, and testing at NVSL has shown the reference antibodies made against A/mallard/Gurjev/263/1982(H14N5) will inhibit hemagglutination of some H4 IAV isolates (data not shown). Therefore, additional H14 IAV isolates, misclassified as H4, might currently exist in other viral repositories.

**Figure 4:**
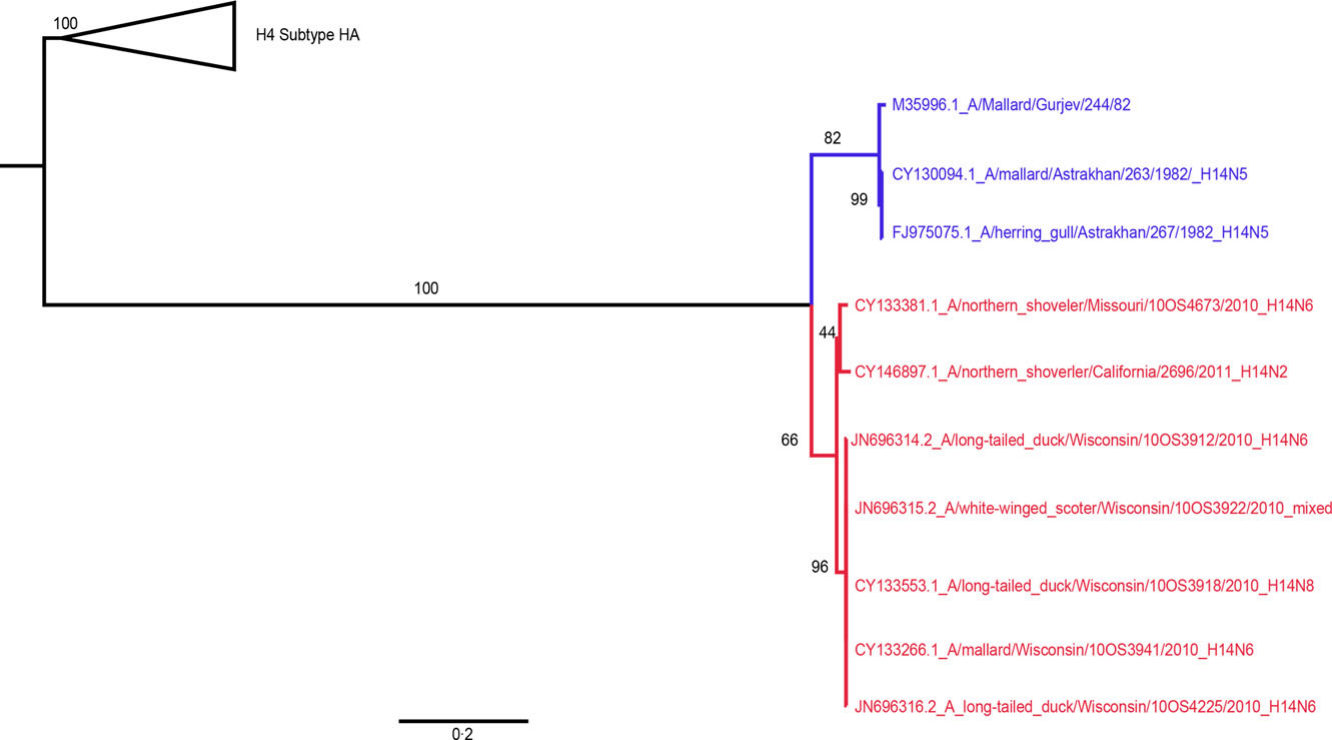
Maximum-likelihood tree with GTR+*Γ* model for the H14 hemagglutinin segments. Included were all North American (red) and Eurasian (blue) publically available nucleotide sequences for the H14 segment.

The H14 tree (Figure4) showed A/northern shoveler/Missouri/10OS4673/2010(H14N6) appears to be a different lineage of H14 IAV than the five Wisconsin H14 isolates, which were all recovered in the same general location within 1 month of each other.^5^ The Wisconsin and Missouri lineages shared 97·5% (554/568) AA identity and had 95·4% and 95·8% AA identity with the original Eurasian H14 isolates recovered from samples collected in 1982, respectively. Additionally, the recently reported A/northern shoveler/California/2696/2011(H14N2)^15^ had 97·4% AA identity with both the Wisconsin and the Missouri lineages. These data indicate that even though the H14 subtype was only recently identified in North America, the H14 IAVs are more genetically diverse than previously thought, possibly due to undetected circulation.

## Conclusions

Finding differences between antigenic and genomic subtyping is not surprising, and each approach contributes valuable information. However, the differences between subtype classification methods delayed the detection of interesting IAV isolates in this study. The recognition of additional H14 and Eurasian-origin H10, N6, and NS genomic segments provides additional evidence for interhemispheric spread of avian IAVs. Reassortment of segments between eastern and western hemisphere avian-origin IAV lineages demonstrates the value of sequencing recovered IAV isolates. In addition, sequencing allows researchers to capture and characterize IAV genetic diversity in the natural reservoir that cannot be fully appreciated with antigen–antibody reactions. Finally, sequencing results serve as indicators for the need to update the IAV reference antisera and strains used in antigenic testing. Further investigations into the cause of the discrepancies between antigenic and genomic subtyping observed in the present study are needed, including the amount of steric inhibition, cross-reactivity between subtypes, non-specific inhibition, and the detection limits of HA titers in HI/NI tests. Continued sequencing of historic avian-origin IAV repositories will be used to address this dynamic and could reveal additional unique IAV diversity.
